# The impacts of COVID-19 on seafood prices in Japan: A comparison between cheap and luxury products

**DOI:** 10.1371/journal.pone.0291395

**Published:** 2023-10-05

**Authors:** Kentaka Aruga, Md. Monirul Islam, Arifa Jannat

**Affiliations:** 1 Graduate School of Humanities and Social Sciences, Saitama University, Sakura-ku, Saitama, Japan; 2 Department of Agricultural Economics, Bangladesh Agricultural University, Mymensingh, Bangladesh; 3 Institute of Agribusiness and Development Studies, Bangladesh Agricultural University, Mymensingh, Bangladesh; University of Economics Ho Chi Minh City, VIET NAM

## Abstract

Like many other countries, the economy and society of Japan have been severely affected by the COVID-19 outbreak, and the fishery sector particularly seafood is no exception. Among seafood, since luxury seafood is more commonly consumed at restaurants it has a higher possibility of getting affected by the pandemic compared with cheap popular seafood for the masses. Considering this motivation, this study investigates the variations in the COVID-19 impact on luxury and cheap seafood prices in the Tokyo Toyosu wholesale market. Using the non-linear autoregressive distributed lag model (NARDL), the study identifies that an increase in the hours of stay-at-home during the initial stage of the pandemic is causing a negative influence on both cheap and luxury seafood prices and that this negative impact was severer in the luxury seafood prices. The study also finds a positive influence from the hours of stay-at-home on some cheap popular seafood like horse mackerel and sardine during the third state of emergency (SOE) where at least most of the elderly people have received their first vaccine dose but the negative impact from the SOE measure remained on luxury seafood. It is evident from the findings that the luxury seafood market is heavily dependent on the restaurant sector, which will likely suffer adverse effects when human mobility is restricted. In the event of a pandemic like COVID-19, policymakers should stabilize the price and provide subsidies to the luxury seafood stakeholders.

## Introduction

With the spread of COVID-19 infections, the global market has plummeted, and the fishing industry, which relies on international trade has been adversely impacted from the beginning. Thus, the COVID-19 pandemic caused a substantial shock to the Japanese economy and the fisheries sector is no exception [1, 2]. Japan is regarded as one of the world’s most important fisheries nations, particularly in terms of seafood production (ranked 8th in marine capture production [3] where seafood serves as one of the most important sources of animal protein for Japanese people [4].

According to the 2020 United States Department of Agriculture (USDA) report, the COVID-19 pandemic caused food service sales in restaurants and hotels to nosedive and tourism to a halt in Japan as public outings have fallen following official requests by the government to remain people in their homes for longer hours [5]. The seafood supply chain was no exception to receive an adverse impact from the pandemic as consumer demand and human mobility declined significantly due to the imposition of a state of emergency (SOE) by the Japanese government [6]. The Tokyo Toyosu wholesale market, which plays the role of the center of Japan’s seafood market experienced a drastic financial fallout from March to April 2020. Both the volume of seafood and prices received negative shocks due to changes in food-taking habits (taking food at home instead of eating outside) as a consequence of human mobility restrictions related to the SOE measures. The report also mentioned that sales of luxury seafood items have dropped significantly, while sales of fish that are more commonly prepared at home have not seen a significant falloff [7].

We assume that the restrictions enforced on human mobility during the SOE reduced the seafood demand, leading to a decrease in market prices. We also expect that the luxury seafood demand is more severely hit by the SOE measures compared to cheap seafood for the masses since this seafood is particularly prepared at restaurants.

To investigate these hypotheses, the present study attempts to examine the variations in the impact of the COVID-19 pandemic on cheap and luxury seafood prices during different states of emergencies from the case of the Tokyo Toyosu wholesale seafood market. This paper is one of the first studies to cover how the changes in the hours of stay at home due to restrictive measures taken during the COVID-19 pandemic have impacted the seafood markets. We believe it contributes to the related literature for understanding how a shock in the demand caused by constraints in human mobility influenced the seafood markets, which provides valuable information for stakeholders involved in the seafood markets and policymakers coping to mitigate the effects of the pandemic on seafood markets.

As COVID-19 also spread out from food products, consuming all forms of meat declined as a precautionary measure, especially undercooked products [8–10]. As a result, COVID-19 had huge impacts on the seafood sector, such as processor closures, shortening of fishing seasons, and disruptions in the production and distribution of seafood, with less impact on frozen products [10]. Although the government would rather choose a voluntary-based request to stay home than legally bind people’s behavior [11], many people in Japan refrained from going out, and the time spent at home increased similarly to other countries taking strict lockdown measures [12]. Thus, the request to stay home had wide-ranging effects on economies in Japan, including lifestyles, and tourism [13, 14]. In previous literature for economic studies, the impact of lockdown policies and COVID-19 on food prices has been discussed in several publications [15–17]. The possibility of the impact of COVID-19 on food demand has also been discussed [18], but there are few empirical studies of the impact on food demand. However, not many studies have analyzed the impact on the seafood market due to the imposition of restrictions at different times. In the case of Japan, where the lockdown was not legally imposed, it is necessary to understand the impact of COVID-19 on the seafood market.

## Literature review

### Theoretical and empirical foundation of the NARDL model

The theoretical framework of the nonlinear autoregressive distributed lag (NARDL) model is based on the introduction of the asymmetric cointegrating regression model and derives the associated asymptotic theory [19]. NARDL is an econometric model used for analyzing the dynamic relationships between variables in time series data, particularly in the context of nonlinearity [19]. The NARDL model extends the traditional autoregressive distributed lag (ARDL) model by incorporating nonlinearities in the relationships between variables [20, 21]. It allows for asymmetric and nonlinear effects, capturing both short-term and long-term dynamics. This makes it suitable for investigating complex relationships that may exhibit nonlinear patterns, such as threshold effects, asymmetries, or asymmetric adjustment speeds. It draws on the idea that economic variables are interconnected and influenced by their past values, as well as the values of other relevant variables. The key components of the NARDL framework include the lagged values of variables, error correction terms, and various nonlinear functions to capture the nonlinearity in the relationships [19]. The model allows for the estimation and testing of the short-term and long-term effects, as well as the direction and magnitude of these effects, considering potential asymmetries. Researchers and economists utilize the theoretical framework of NARDL to analyze and understand the dynamics between variables in a time series setting. Several studies have employed the NARDL model to analyze the impact of various shocks on economic variables, providing a solid theoretical foundation for its application in this research.

For instance, Aruga [22] used the NARDL model to investigate the effects of the COVID-19 pandemic on the Tokyo wholesale tuna market, and the findings showed that an increase in the hours of stay-at-home during the first SOE had an adverse impact on all three tuna prices in the long run. Jannat et al. [23] applied the NARDL model to examine the impact of international markets on imported and exported non-cereal crops in Bangladesh. They found evidence of asymmetric effects: the world potato and rapeseed prices led to an increase in Bangladesh potato and rapeseed prices when they were increasing. Furthermore, Van Treeck [24] has employed the NARDL model in his analysis of asymmetric wealth effects on United States (US) consumption and found that liquidity constraints and loss-aversion can be reconciled inter-temporally, with the former dominating in the short-run and the latter in the long-run. Delatte and López-Villavicencio [25] applied the NARDL technique in their analysis of long-run asymmetries in the pass-through from exchange rates to consumer prices in developed economies. These studies emphasized the importance of capturing such nonlinearities and asymmetries to gain a comprehensive understanding of the relationship between variables. Overall, the theoretical and empirical framework of NARDL provides a systematic approach for modeling and analyzing nonlinear relationships in time series data, enabling researchers to explore complex dynamics that go beyond the linear assumptions of traditional econometric models.

Furthermore, there are previous studies investigating the impacts of human mobility changes related to COVID-19 on markets other than seafood such as those examining the effects on Japanese fuel prices [26], levels of CO_2_ emissions [27], agricultural commodity markets in developing economies [28], and food prices in Europe [29], seafood safety and human health [30], and policy response to COVID-19 in the ASEAN region [31]. For a study examining the impact of the pandemic on Japanese fisheries, Sugimoto et al. [32] surveyed stakeholders of Japanese fisheries including scientists involved in the seafood business and fisheries sector to reveal negative changes in 2020 sales compared to a year earlier caused by the pandemic. However, this study did not use the actual market price of seafood to find out how the pandemic caused impacts on the Japanese seafood market. A report from Global Fishing Watch is also supporting this demand-oriented shock in the Japanese seafood market, showing that fishing hours did not decline during the COVID-19 pandemic in Japan, rather they increased [33]. Thus, the price decline in Japan during the pandemic is similar to the case of fishermen’s impoverishment due to a bumper harvest caused by the decline in demand. Errhalt [33] discussed how Japanese fisheries had to sell fish when domestic demand decreased during the pandemic.

### Research gap

Considering the unique nature of the COVID-19 pandemic and its potential impact on seafood prices in Japan, it is reasonable to expect that the shocks experienced in this context may exhibit non-linear and asymmetric effects. The disruption in supply chains (sales volume), changes in the number of COVID-19 cases, and people staying at home during the different state of emergency periods have likely influenced seafood prices differently depending on the product category (cheap vs. luxury). To the best of our knowledge, no previous study has specifically employed the NARDL model to investigate the impacts of COVID-19 on seafood prices in Japan, particularly focusing on the comparison between cheap and luxury products. By adopting the NARDL model, this research aims to fill this gap and contribute to the existing literature by providing a nuanced understanding of the dynamics between COVID-19 shocks and seafood prices, capturing both short-term and long-term effects as well as any potential asymmetries.

To bridge this gap, the present study tests how the cheap and luxury seafood prices at different SOE periods during 2020–2021 in Japan have been influenced by the SOE measures taken to restrict human mobility. Hence, this is one of the pioneer studies to identify how daily prices of luxury and cheap seafood are affected by changes in human mobility during the COVID-19 pandemic. The research aims to answer two primary questions:

1. How does COVID-19 affect seafood consumption patterns at different SOE periods?
2. What are the symmetric and asymmetric effects of the COVID-19 pandemic on the seafood market?

Following this, the first contribution of the study is that the study results provide imperative information for the stakeholders of the seafood market to prepare and deal with shocks that could occur when a similar restriction controlling human mobility is imposed in the future to deal with a crisis like the COVID-19 pandemic. The second contribution is that by comparing the effects (short-run and long-run) of the pandemic on cheap and luxury seafood, the study can provide some evidence on which type of seafood might be more vulnerable to a measure restricting human mobility during the pandemic- whereas early literature on pandemics largely ignored this area.

## Materials and methods

### The four states of emergency and the luxury and cheap seafood markets

The study focuses on the effect of the COVID-19 pandemic on fresh seafood traded at the Toyosu Wholesale Fish Market since this market is one of the world’s largest wholesale seafood markets, replacing the former Tsukiji Fish Market. Another reason for investigating the Tokyo seafood market is that Tokyo is known as the fourth most expensive metropolis in the world and has the highest cumulative number of COVID-19 cases among all Japanese cities [34].

**Fig 1:**
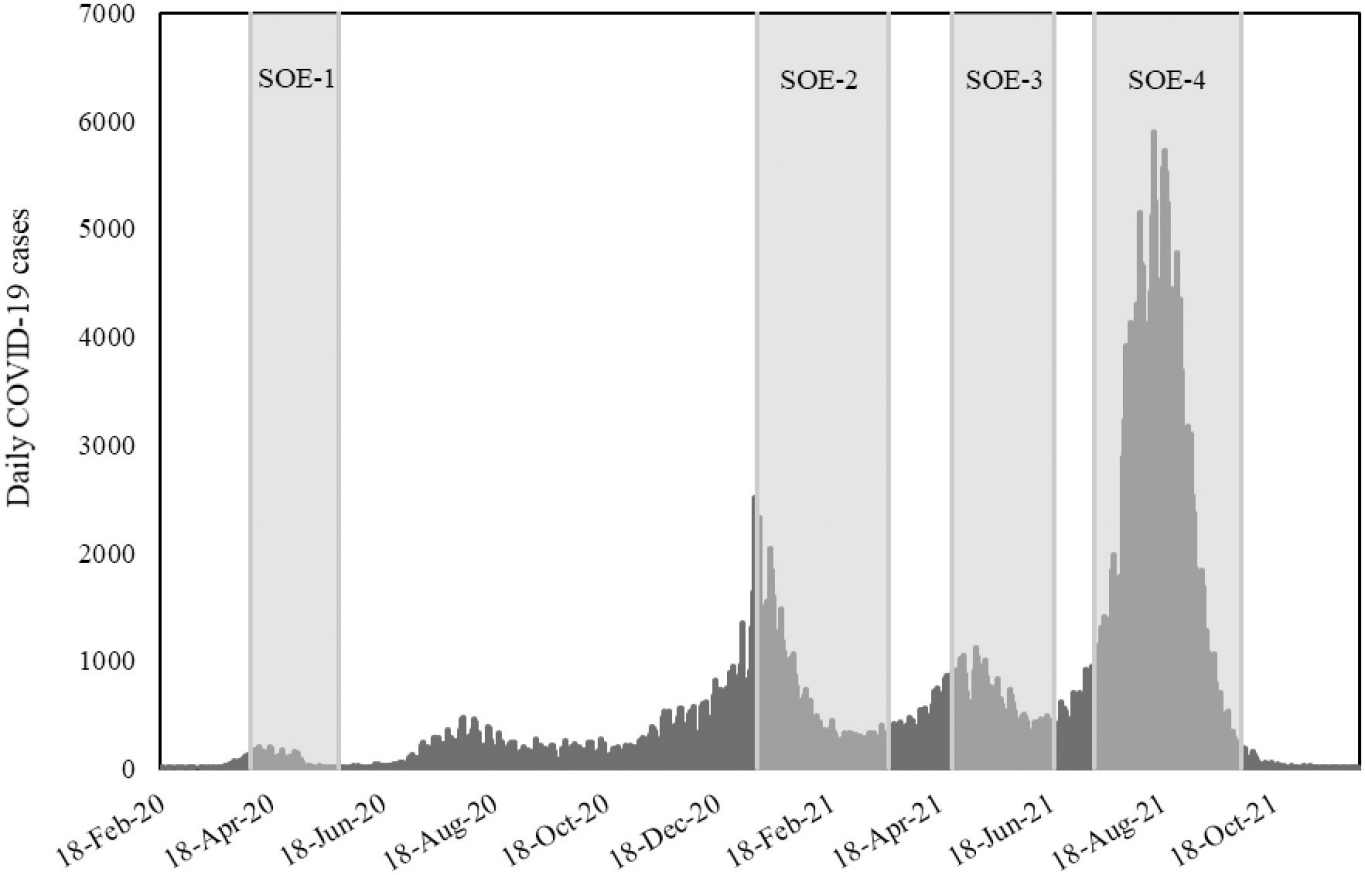
Daily COVID-19 cases detected in Tokyo and the SOE periods. Source: Tokyo Metropolitan Government [45].

As seen in Fig 1, the first wave of COVID-19 cases in Tokyo, Japan began in March 2020. Since then, the SOE has been announced four times by the Tokyo Metropolitan Government to reduce the number of COVID-19 cases.

Thus, we picked daily prices per kilogram and daily total trade volumes (kg) of horse mackerel (*aji*), sardine (*iwashi*), and mackerel (*saba)* to represent the prices for cheap popular seafood. In Japan, the three seafood have been traditionally and commonly consumed by households and are known as *taishu gyo*, which means major popular cheap fish for the masses. As a comparison to the cheap seafood, we also chose daily prices of the three-luxury seafood, Japanese tiger shrimp (*kuruma ebi*), channel rockfish (*kinki or kichiji*), and sea urchin (*uni*). These seafood products are traded in the same category as the cheap seafood examined in this study at the Toyosu wholesale market, fresh seafood, and had a higher average price within the fresh seafood category and are well known as luxury seafood. There are two types of trades in the Toyosu market; negotiation and auction. However, only the tiger shrimp and sea urchins were traded under the auction system so the prices of all other products (horse mackerel, sardine, mackerel, and rockfish) are based on the negotiated trade. In addition, for all products, the daily medium price is used when this price is available, and the average of the high and low prices is applied when the medium price was missing (Table 1).

**Table 1:** Description of variables.

Variable	Description	Measurement unit	Sources
HMP	Horse mackerel price	Price/kg (JPY)	[46]
HMV	Horse mackerel volume	Volumes (kg)	
SP	Sardine price	Price/kg (JPY)	
SV	Sardine volume	Volumes (kg)	
MP	Mackerel price	Price/kg (JPY)	
MV	Mackerel volume	Volumes (kg)	
SP	Shrimp price	Price/kg (JPY)	
SV	Shrimp volume	Volumes (kg)	
RP	Rockfish price	Price/kg (JPY)	
RV	Rockfish volume	Volumes (kg)	
SUP	Sea urchin price	Price/kg (JPY)	
SUV	Sea urchin volume	Volumes (kg)	
Home	The number of visitors to residential areas has changed compared to baseline days (the median value for the 5 weeks from 3 January to 6 February 2020). This index is smoothed to a rolling 7-day average.	Percentage change	[36]
COVID	Daily number of COVID-19 cases deteceted in Tokyo	Number of patients	[45]

Moreover, we chose the sales prices of different seafood products as the dependent variable and the residential index, sales volume, and COVID-19 parameters as independent variables for our model for several reasons. To make informed decisions, businesses and policymakers must understand the factors influencing seafood prices at different SOE periods. It is possible to gain insight into how these factors affect price determination not only in the short run, but also in the long run, by analyzing the relationship between sales prices and people staying at home (residential index), sales volume, and the number of COVID-19 cases. Moreover, in economic models, sales volume is often used to represent demand [35]. The impact of demand fluctuations on seafood prices can be assessed by incorporating sales volume as an independent variable. On the other hand, the residential index can represent consumer behavior changes, which can offer insights into shifts in demand and supply dynamics during the pandemic. Additionally, COVID-19 influences people’s movements and may capture changes in market conditions, such as disruptions in the supply chain or shifts in consumer preferences due to the pandemic. Studying the relationship between prices, the residential index, sales volume, and COVID-19 variables can provide insights into pricing strategies’ effectiveness. With the residential index, COVID-19, and sales volume as independent variables in a NARDL model, we can provide comprehensive insight into market dynamics, pricing strategies, and the impact of external events such as COVID-19 on seafood prices.

The periods when Tokyo was under the SOE are summarized in Table 2. Since the Tokyo government enacted a semi-SOE where the restaurants’ business hours were limited to 9:00 pm for some of the SOEs like the third SOE (12–24 April 2021 and 21 June–11 July 2021), we anticipate that the impact of SOE on human mobility will be sustained for a given period before and after the SOE. To capture these pre- and post-effects, we designated our SOE periods as those four weeks before and after the SOE and our SOE periods include these pre and post four weeks besides the actual days when the SOE was enforced.

**Table 2:** Tokyo’s state of emergency periods during 2020–2021.

SOEs	4 weeks before	Starting	Ending	4 weeks after
First	10-Mar-2020	7-Apr-2020	25-May-2020	22-Jun-2020
Second	11-Dec-2020	8-Jan-2021	21-Mar-2021	17-Apr-2021
Third	28-Mar-2021	25-Apr-2021	20-Jun-2021	17-Jul-2021
Fourth	15-Jun-2021	12-Jul-2021	30-Sep-2021	27-Oct-2021

Although the SOE measure conducted in Tokyo was not a harsh lockdown regulation with severe penalties, the business hours of restaurants and bars were regulated from 5 am to 8 pm, large public events were prohibited, and the Tokyo metropolitan government requested companies to have their employees work from home. To capture the changes in human mobility during the SOE periods, we obtained an index measuring the daily changes in the hours of stay in the residential area for Tokyo. The “residential” index obtained from the Google Global Mobility Report is the one used for this purpose, representing the changes in the Tokyo residents’ number of hours spent in the residential area relative to the baseline day defined as the median value between 3 January and 6 February 2020 [36].

Since the SOE restriction affected the restaurants the most, we expect that the effect of reduction in human mobility related to SOE is different between cheap popular seafood mostly consumed at homes and luxury seafood particularly prepared in restaurants [7].

### Econometric model

Since the study wanted to identify both the short-run and long-run impacts of the human mobility changes on seafood prices, we applied the auto-regressive distributed lag (ARDL) model proposed by Pesaran et al. [20]. One of the primary motivations for using the ARDL model is that it can provide valid results even with the sample size is relatively small and can prevent omitted variables and auto-correlation problems [20]. Within the ARDL framework, we used the NARDL model developed by Shin et al. [19] to capture the asymmetric adjustment patterns regarding the positive and negative shocks of human mobility on prices.

Based on the prior model developed for investigating the effects of human mobility on the fuel markets [26], the impact of variations in human mobility during the COVID-19 pandemic on cheap and luxury seafood market prices was examined using the following model:

$$price_{t}=a+b_{1}home_{t}+b_{2}volume_{t}+b_{3}COVID+\varepsilon_{t}$$ (1)

where *price*_*t*_ is either one of the daily seafood prices investigated in this study. Similarly, *home*_*t*_ is the stay-at-home index reflecting the changes in human mobility during the SOE periods and *volume*_*t*_ is the total daily volume of trades at the Toyosu market for each of the six seafood products, and *COVID* is the daily COVID-19 cases in Tokyo. *b*_1_, *b*_2_ and *b*_3_ are the coefficient of the variables.

Before employing the econometric approaches, we conducted both conventional stationarity tests and those with the effect of a structural break in the time series data. For this purpose, we employed the Augmented Dickey-Fuller (ADF), the Zivot & Andrews (ZA) [37], and the Kwiatkowski–Phillips–Schmidt–Shin (KPSS) tests. Table 3 illustrates the results of these unit root tests. The outcome ensures that none of the underlying series is stationary at the second difference, and all variables are at least stationary at their levels or first differences.

**Table 3:** Unit root tests.

Variables	SOE 1												SOE 2											
	Levels						First differences						Levels						First differences					
	ADF		ZA		KPSS		ADF		ZA		KPSS		ADF		ZA		KPSS		ADF		ZA		KPSS	
HMP	-3.98	**	-4.71	**	0.13	*	-7.38	***	-4.71	***	0.07		-6.24	***	-6.62	***	0.04		-10.44	***	-10.53	***	0.08	
HMV	-4.84	***	-5.42	***	0.08		-12.26	***	-12.31	***	0.02		-6.31	***	-6.68	***	0.06		-12.44	***	-12.42	***	0.50	***
SDP	-6.17	***	-7.70	***	0.13	*	-9.89	***	-10.45	***	0.50	***	-3.03		-9.10	***	0.16	**	-10.92	***	-11.80	***	0.14	*
SDV	-9.42	***	-10.09	***	0.28	***	-9.34	***	-10.28	***	0.50	***	-6.78	***	-7.05	***	0.09		-10.13	***	-10.47	***	0.06	
MP	-7.03	***	-8.71	***	0.17	**	-13.92	***	-14.83	***	0.28	***	-6.23	***	-8.34	***	0.32	***	-8.06	***	-8.23	***	0.14	*
MV	-6.88	***	-7.35	***	0.10		-8.57	***	-8.80	***	0.18	**	-4.85	***	-6.32	***	0.22	***	-14.03	***	-14.02	***	0.14	*
SHP	-1.80		-8.31	***	0.23	***	-9.88	***	-10.22	***	0.02		-4.98	***	-5.27	***	0.13	*	-11.36	***	-11.34	***	0.25	***
SHV	-4.65	***	-5.71	***	0.11		-9.99	***	-10.30	***	0.39	***	-4.55	***	-5.57	***	0.18	**	-8.24	***	-8.33	***	0.20	**
RP	-7.71	***	-7.86		0.04		-7.39	***	-9.84	***	0.29	***	-5.59	***	-5.72	***	0.10		-13.31	***	-13.47	***	0.20	**
RV	-7.49	***	-8.53		0.17	**	-7.29	***	-10.63	***	0.01		-8.91	***	-9.01	***	0.04		-12.96	***	-12.95	***	0.02	
UP	-5.85	***	-6.89	***	0.16	**	-9.33	***	-9.39	***	0.15	*	-3.94	**	-7.37	***	0.25	***	-8.77	***	-8.96	***	0.24	***
UV	-2.66		-9.91	***	0.14	*	-8.24	***	-8.32	***	0.05		-9.03	***	-10.16	***	0.07		-8.71	***	-8.73	***	0.02	
Home	-0.65		-3.68		0.28	***	-4.16	***	-8.79	***	0.50	***	-6.37	***	-8.45	***	0.22	***	-10.47	***	-10.52	***	0.28	***
	SOE 3												SOE 4											
HMP	-4.08	***	-4.23		0.07		-10.67	***	-10.67	***	0.07		-4.27	***	-4.49	*	0.05		-10.82	***	-11.23	***	0.03	
HMV	-5.15	***	-5.51	***	0.06		-10.94	***	-10.98	***	0.12	*	-5.63	***	-5.68	***	0.04		-12.12	***	-36.61	***	0.12	*
SDP	-6.59	***	-6.82	***	0.11		-9.23	***	-9.47	***	0.13	*	-7.93	***	-9.64	***	0.31	***	-10.25	***	-10.28	***	0.15	**
SDV	-6.28	***	-6.50	***	0.11		-8.98	***	-11.47	***	0.16	**	-5.94	***	-6.83	***	0.21	**	-7.91	***	-9.89	***	0.50	***
MP	-7.60	***	-8.11	***	0.16	**	-11.70	***	-11.81	***	0.12		-7.01	***	-7.07	***	0.06		-8.58	***	-10.09	***	0.50	***
MV	-6.97	***	-7.17	***	0.11		-8.56	***	-8.80	***	0.32	***	-6.87	***	-7.49	***	0.14	*	-9.67	***	-9.93	***	0.50	***
SHP	-4.74	***	-5.42	***	0.18	**	-11.95		-12.36	***	0.15	*	-6.14	***	-6.41	***	0.08		-9.66	***	-13.41	***	0.12	
SHV	-9.09	***	-9.28	***	0.08		-18.30		-18.29	***	0.12	*	-6.08	***	-7.17	***	0.22	***	-8.13	***	-10.92	***	0.12	*
RP	-9.36	***	-9.83	***	0.10		-8.31	***	-8.36	***	0.13	*	-9.09	***	-9.23	***	0.20	**	-9.21	***	-11.97	***	0.11	
RV	-8.75	***	-9.02	***	0.05		-11.79	***	-12.24	***	0.06		-3.96	**	-8.31	***	0.21	**	-9.34	***	-9.62	***	0.33	***
UP	-6.07	***	-6.11	***	0.10		-7.49	***	-12.19	***	0.19	**	-5.38	***	-7.05	***	0.20	**	-12.25	***	-10.29	***	0.15	***
UV	-5.37	***	-5.49	***	0.06		-7.14	***	-7.31	***	0.06		-4.37	***	-4.36	*	0.06		-11.38	***	-11.69	***	0.07	
Home	-2.03		-9.89	***	0.25	***	-11.67	***	-11.63	***	0.19	**	-1.90		-4.50	*	0.35	***	-12.57	***	-12.04	***	0.08	

Note:

***, **, and * denote significance at the 1%, 5%, and 10% levels, respectively. HMP, SDP, MP, SHP, RP, UP, and HMV, SDV, MV, SHV, RV, UV are the prices and volumes traded for horse mackerel, sardine, mackerel, channel rockfish, kuruma shrimp, and sea urchin, respectively.

After confirming the unit root result, the NARDL estimation was performed. Initially, the existence of long-run relationships among the price, volume, and home variables was tested through the NARDL bound test for cointegration [38]. The statistically appropriate lag lengths for the NARDL were determined based on the Akaike Information Criterion (AIC).

The NARDL model is based on the following ARDL unrestricted error correction model:

$$\begin{array}{l} \Delta price_{t}=a+b_{1}price_{t-1}+b_{2}home_{t-1}+b_{3}volume_{t-1}+\overset{p}{\underset{i=1}{\sum }}b_{4i}\Delta price_{t-i}\\ +\overset{q}{\underset{i=0}{\sum }}b_{5i}\Delta home_{t-i}+\overset{r}{\underset{i=0}{\sum }}b_{6i}\Delta volume_{t-i}+b_{7}COVID+\varepsilon_{t} \end{array}$$ (2)

where *p* is the lag of the dependent variable, and *q* and *r* are that of the independent variables.

Finally, based on Eq (2), the NARDL model was applied to estimate the relationship presented in Eq (1). For applying the NARDL, the volume and home variables were decomposed into positive and negative cumulative sums. However, the reason to set the lags of the positive and negative partial sums is the same because the variables that we considered are split into negative and positive parts. Therefore, it should be the same number of lags [19].

The positive and negative shocks of changes in the trade volume and hours spent at home are defined below:

$$S_{t}^{+}=\overset{t}{\underset{i=1}{\sum \;}}\Delta S_{i}^{+}=\overset{t}{\underset{i=1}{\sum \;}}\text{max}\left(\Delta S_{i},0\right),\;S_{t}^{-}=\overset{t}{\underset{i=1}{\sum }}\;\Delta S_{i}^{-}=\overset{t}{\underset{i=1}{\sum }}\;\text{min}\left(\Delta S_{i},0\right)$$ (3)

where *S* represents the endogenous variables of our interest (*home* or *volume*), and $S_{t}^{+}$ and $S_{t}^{-}$ denote the partial sums of positive and negative changes of these variables.

Based on the decomposition in Eq (3), the volume and home in Eq (2) are replaced by the positive and negative partial sums of these variables. Using these variables, the NARDL unrestricted error correction model was estimated under the following equation:

$$\begin{array}{l} \Delta price_{t}=a+b_{1}price_{t-1}+b_{2}home_{t-1}^{+}+b_{3}home_{t-1}^{-}+b_{4}volume_{t-1}^{+}+\\ b_{5}volume_{t-1}^{-}+\overset{p}{\underset{i=1}{\sum }}b_{6i}\Delta price_{t-i}+\overset{q}{\underset{i=0}{\sum }}\left(\kappa_{i}^{+}\Delta home_{t-i}^{+}+\kappa_{i}^{-}\Delta home_{t-i}^{-}\right)+\\ \overset{r}{\underset{i=0}{\sum }}\left(\lambda_{i}^{+}\Delta volume_{t-i}^{+}+\lambda_{i}^{-}\Delta volume_{t-i}^{-}\right)+b_{7}COVID+\varepsilon_{t} \end{array}$$ (4)

The Breusch–Godfrey test for autocorrelation [39, 40] and the Breusch & Pagan [41] test for heteroskedasticity were used to determine whether the models had serial correlations and heteroskedasticity issues. None of our models featured some serial correlation issue under the 5% significance threshold, as shown by the Breusch–Godfrey (BG) test findings in Table 4. Based on the 1% significance threshold, the Breusch-Pagan-Godfrey (BPG) test indicated that most of our models are homoscedastic, except shrimp at the SOE-1 and the Mackerel (under SOE-2 & SOE-3) model, which had heteroscedasticity. To address the issues of serial correlation and heteroscedasticity, we estimated the ARDL model coefficients using the Newey–West heteroscedasticity and autocorrelation corrected (HAC) standard errors. The cumulative sum (CUSUM) and cumulative sum square (CUSUM square) tests were also used to assess the stability of the NARDL models. Most of the models except for the sardine in the SOE-2 satisfied the stability test (see S1 Fig). EViews 12 was used to run the NARDL model.

**Table 4:** Serial correlation and heteroskedasticity tests.

**Category**		**SOE 1**			**SOE 2**		
		**BG F-stat**.	**BPG F-stat**.		**BG F-stat**.	**BPG F-stat**.	
Cheap	Horse mackerel	1.218	0.326		0.499	2.138	*
	Sardine	0.241	1.432		1.511	1.535	
	Mackerel	2.015	1.048		0.320	2.574	***
Luxury	Shrimp	0.587	2.914	***	1.014	0.587	
	Rockfish	0.378	0.561		0.350	0.971	
	Sea urchin	0.431	1.605		1.008	1.112	
		**SOE3**			**SOE 4**		
		**BG F-stat**.	**BPG F-stat**.		**BG F-stat**.	**BPG F-stat**.	
Cheap	Horse mackerel	0.555	1.274		0.712	1.105	
	Sardine	1.024	2.052	**	1.542	1.085	
	Mackerel	0.486	2.343	***	0.442	1.556	
Luxury	Shrimp	0.849	0.934		0.107	1.055	
	Rockfish	0.795	1.227		1.089	1.170	
	Sea urchin	0.050	1.102		0.303	1.623	*

Note:

***, **, and * denote significance at the 1%, 5%, and 10% levels, respectively.

## Results and discussions

**Fig 2:**
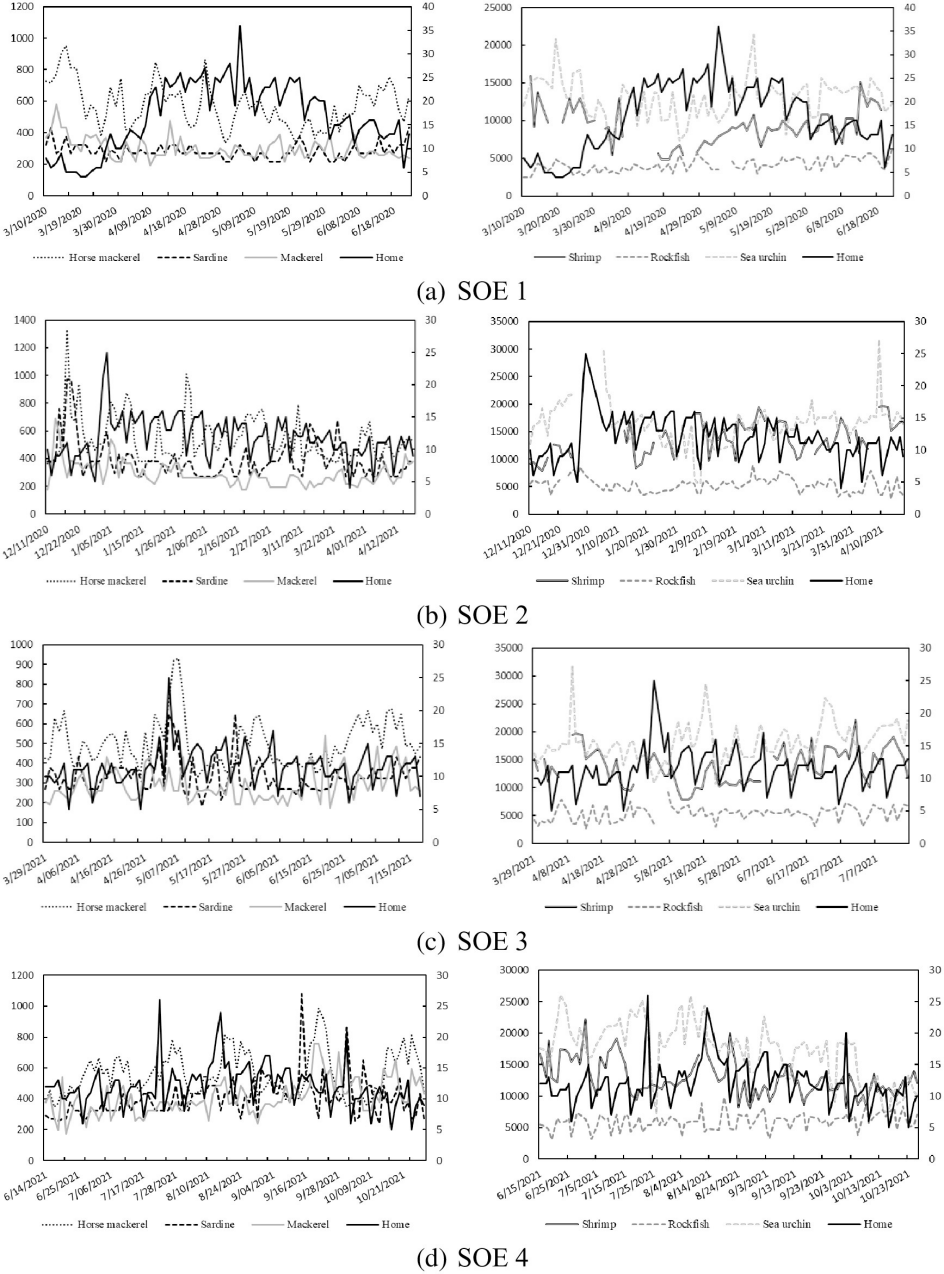
Tokyo Toyosu wholesale fish prices and the stay-at-home index. Note: the left and right vertical axes denote the price per kilogram in JPY and the percentage change in hours of stay-at-home of Tokyo citizens, respectively.

Fig 2 illustrates the plots of the daily cheap and luxury seafood prices investigated in this study along with the daily changes in the stay-at-home (residential) index during the four SOE periods. It is notable from the figure that average prices per kilogram of luxury seafood are mostly over twentyfold of the cheap popular seafood products. It is also discernible that the stay-at-home index remained above the 15% line for a longer time in the first SOE period compared with the other three SOE periods suggesting that the SOE restrictions were more effective in the first SOE.

Table 5 summarizes the descriptive statistics of the price and trade volume data used in the study. our modeled variables are shown in Table 5. It is apparent from the table that except for horse mackerel and mackerel prices, the mean prices of the first SOE period are all lower than those of the second to fourth SOE periods, and even the mean horse mackerel and mackerel prices in the fourth SOE are higher than the first SOE. This is likely indicating that the effect of the SOE restriction on human mobility weakened as time passed by and that the demand for seafood became higher after the second SOE compared with the first SOE. The standard deviation of the prices was also all higher in the fourth SOE than the first SOE implying that the market became more volatile in the fourth SOE.

**Table 5:** Descriptive statistics.

SOE	Stat.	HMP	HMV	SDP	SDV	MP	MV	SHP	SHV	RP	RV	UP	UV
**1** ^**st**^	Mean	575.1	43920.1	284.0	38462.5	299.7	31616.9	9278.5	1281.1	4082.5	1248.2	12982.3	1978.7
	Median	567	44095.5	270	38073.5	281	31202	9180	1254	4050	1160	13538.25	1956.5
	Max	954	83750	432	65066	583	51500	15930	2007	6210	3427	21420	3433
	Min	333	9081	216	19109	194	16392	4860	624	2430	242	7330.5	899
	Std. Dev.	140.81	15233.38	47.09	9210.95	69.83	7076.46	2414.89	310.78	865.44	660.25	2684.32	654.46
	Skewness	0.48	0.10	0.82	0.33	1.30	0.22	0.40	0.10	0.25	0.92	0.12	0.23
	Kurtosis	2.72	2.84	4.23	3.15	5.36	3.26	3.11	2.76	2.26	3.61	4.07102	2.013355
	No. Obs.	74		74		74		63		73		74	
**2** ^**nd**^	Mean	542.9	44742.6	387.9	21866.4	287.2	39112.5	13814.2	1535.0	5299.6	997.9	16204.8	2397.5
	Median	486	44481	378	21877	259	36587	13770	1453	5400	950.5	16416	2321
	Max	1332	104526	972	72972	691	81191	19710	3261	8910	3289	31752	5070
	Min	364.5	4079	270	1383	173	4894	7830	708	2700	37	5670	432
	Std. Dev.	165.47	17636.68	135.61	10352.07	88.37	13132.34	3036.73	386.04	1357.79	549.42	3851.63	837.39
	Skewness	1.80	0.20	2.29	1.34	1.78	0.23	0.01	2.19	0.42	1.25	0.56	0.36
	Kurtosis	7.68	3.51	9.43	8.87	7.74	3.35	2.12	10.07	2.67	5.97	7.21	3.24
	No. Obs.	93		93		93		75		92		88	
**3** ^**rd**^	Mean	500.5	56168.4	333.6	29558.5	290.2	27026.0	13819.3	1445.5	5353.5	1195.3	18235.0	2634.5
	Median	483.375	60263.5	324	27789	259	25800	13905	1442	5400	1130	17718.75	2511
	Max	931.5	87623	648	69771	540	44549	22140	1924	9180	3043	31752	4606
	Min	330.75	11035	184	7531	173	16811	7830	906	2700	385	10678.5	811
	Std. Dev.	122.50	18152.05	86.53	12017.44	83.00	5805.88	3232.84	180.99	1355.77	470.06	3461.92	780.43
	Skewness	1.16	-0.41	1.60	1.06	0.94	0.60	0.24	-0.05	0.05	1.21	1.06	0.21
	Kurtosis	4.72	2.33	6.34	4.41	3.12	3.05	2.28	3.45	2.51	5.68	5.56	2.42
	No. Obs.	80		80		80		74		79		80	
**4** ^**th**^	Mean	558.4	46668.7	402.9	21581.1	407.7	23213.2	12861.3	1312.6	6022.4	976.8	17244.9	2290.6
	Median	526.5	47140	378	15884	378	22796.5	12312	1321	6210	941	18162	2272
	Max	985.5	84049	1080	69771	756	42479	22140	1889	9720	2273	26010	3915
	Min	345.5	11276	238	2559	173	8519	7992	750	3078	142	7114.5	754
	Std. Dev.	143.56	17822.43	125.49	15311.17	115.33	6648.13	2920.85	222.37	1306.98	408.50	4635.93	788.76
	Skewness	0.67	-0.01	2.26	1.04	0.78	0.22	0.78	-0.07	-0.12	0.46	-0.40	0.27
	Kurtosis	2.80	2.27	11.89	3.43	3.78	3.01	3.30	3.02	3.06	3.32	2.53	2.16
	No. Obs.	94		94		94		93		95		95	

Note: HMP, SDP, MP, SHP, RP, UP, and HMV, SDV, MV, SHV, RV, UV are the prices (kg/JPY) and volumes (kg) traded for horse mackerel, sardine, mackerel, channel rockfish, kuruma shrimp, and sea urchin, respectively.

To test for cointegration relationships among the test variables, we conducted the NARDL bound test for cointegration. Table 6 shows that the F-statistics is larger than the upper bounds at the 5% significance level for all seafood models except in the sardine SOE-1 (3.21) and sea urchin SOE-2 (2.95) periods. Thus, most of our seafood models had cointegration relationships indicating the existence of a long-run relationship among price, volume, and stay-at-home index. Next, we estimated the NARDL long-run coefficients. The estimated results are presented in Table 7.

**Table 6:** NARDL bound tests.

Category		SOE1		SOE2		SOE3		SOE4	
		F-stat.		F-stat.		F-stat.		F-stat.	
Cheap	Horse mackerel	12.69	***	13.58	***	15.00	***	9.94	***
	Sardine	3.21		6.14	***	11.68	***	13.24	***
	Mackerel	14.72	***	9.92	***	4.57	**	11.60	***
Luxury	Shrimp	9.79	***	5.33	***	7.84	***	6.48	***
	Rockfish	10.62	***	9.28	***	16.88	***	11.94	***
	Sea urchin	12.91	***	2.95		7.43	***	9.80	***

Note:

***, **, and * denote significance at the 1%, 5%, and 10% levels, respectively.

**Table 7:** NARDL long-run coefficients.

	Cheap									Luxury								
	Horse mackerel			Sardine			Mackerel			Shrimp			Rockfish			Sea urchin		
**SOE 1**	**Coef**.		**t-stat**.	**Coef**.		**t-stat**.	**Coef**.		**t-stat**.	**Coef**.		**t-stat**.	**Coef**.		**t-stat**.	**Coef**.		**t-stat**.
Volume+	-0.008	***	-8.717	-0.006	*	-1.898	-0.003	**	-2.224	3.994	***	4.296	-0.644	***	-3.772	-1.421	**	-2.229
Volume-	-0.007	***	-7.365	-0.006	*	-1.975	-0.002		-1.319	3.007	***	3.359	-0.688	***	-3.710	-0.603		-0.793
Home+	-7.169	**	-2.638	-6.091	*	-1.781	0.219		0.420	-165.322	***	-3.286	-19.491		-1.197	-85.746	*	-1.847
Home-	-10.410	**	-2.573	-7.433		-1.582	-1.339		-1.657	-100.843	*	-1.990	-28.169		-1.642	-280.601	***	-2.751
constant	724.080	***	23.187	211.042	***	4.338	361.230	***	13.054	9773.697	***	9.428	3183.374	***	9.820	12863.040	***	11.617
**SOE 2**	**Coef**.		**t-stat**.	**Coef**.		**t-stat**.	**Coef**.		**t-stat**.	**Coef**.		**t-stat**.	**Coef**.		**t-stat**.	**Coef**.		**t-stat**.
Volume+	-0.007	***	-4.760	-0.005		-1.650	-0.004	*	-1.938	6.954		1.518	-0.186		-0.556	0.233		0.217
Volume-	-0.007	***	-4.668	-0.004		-1.417	-0.003	**	-2.248	2.020		0.628	0.259		0.830	1.808		0.947
Home+	-9.373		-1.633	1.502		0.152	-3.126		-1.154	-458.260		-1.607	-331.085	***	-3.420	-2774.435		-1.481
Home-	-11.042		-1.504	0.361		0.038	-5.445		-1.387	-28.684		-0.130	-407.085	***	-3.902	-3309.857		-1.362
constant	447.080	***	5.355	674.763	***	4.599	276.829	***	7.463	14197.820	***	8.525	5263.942	***	6.447	17171.330	***	4.864
**SOE 3**	**Coef**.		**t-stat**.	**Coef**.		**t-stat**.	**Coef**.		**t-stat**.	**Coef**.		**t-stat**.	**Coef**.		**t-stat**.	**Coef**.		**t-stat**.
Volume+	-0.004	***	-6.087	-0.001		-0.927	-0.010	*	-1.916	-2.324		-0.591	-0.168		-0.363	-2.721	*	-1.688
Volume-	-0.006	***	-8.656	-0.002	***	-2.980	-0.011	**	-2.383	-4.363		-0.920	-0.254		-0.683	-3.123	*	-1.901
Home+	16.948	***	3.456	29.027	***	4.169	-17.625	***	-2.800	-1027.632	***	-3.958	-14.658		-0.164	218.090		0.889
Home-	27.182	***	4.052	34.070	***	4.490	-15.679	**	-2.243	-908.512	**	-2.589	-9.126		-0.124	334.161		1.246
constant	278.963	***	5.606	146.254	***	3.980	347.507	***	5.740	21351.770	***	10.292	4141.456	***	5.314	16200.340	***	9.098
**SOE 4**	**Coef**.		**t-stat**.	**Coef**.		**t-stat**.	**Coef**.		**t-stat**.	**Coef**.		**t-stat**.	**Coef**.		**t-stat**.	**Coef**.		**t-stat**.
Volume+	-0.006	***	-5.973	-0.001		-1.356	-0.008	**	-2.615	-0.228		-0.093	0.012		0.062	-2.183		-1.375
Volume-	-0.006	***	-6.959	-0.003	***	-2.648	-0.010	***	-3.167	2.693		1.135	-0.039		-0.215	-2.919	*	-1.825
Home+	-0.856		-0.152	3.261		0.379	-0.407		-0.098	0.456		0.002	-48.465		-0.880	-66.542		-0.239
Home-	0.789		0.146	6.454		0.794	3.430		1.041	-134.665		-0.977	-51.550		-0.855	254.641		1.156
constant	421.039	***	11.477	261.545	***	8.078	282.314	***	9.116	16070.570	***	14.534	5553.731	***	15.950	24945.380	***	7.333

Note:

***, **, and * denote significance at the 1%, 5%, and 10% levels, respectively.

During the first SOE period, the statistically significant results of the stay-at-home index indicate that both the positive and negative shocks in the hours of stay-at-home have negative impacts on seafood prices. These results imply that the SOE measures conducted in the first SOE period had adverse impacts on the seafood price. This result is consistent with Aruga [22] finding a negative impact on tuna prices during the first SOE period. As for the variable *volume*, the prices of horse mackerel, shrimp, and rockfish are significantly influenced by the trade volume at 1% and 5% levels, both positively and negatively. It is evident that both positive and negative changes in trade volume affect the price of cheap and luxury fish due to the closure of restaurants. Similar findings were observed in a report that mentioned bluefin tuna, sea urchins, black throat seaperch, and tiger prawns are among the seafood essentials for Tokyo-style sushi that have seen their prices fall. All of these have seen price drops by more than 30%, leaving fish pros stunned [42]. This is likely related to the reduced seafood trade in 2020 since the supply chain for seafood has been affected by the suspension of international transportation by air and sea [43].

For the second SOE period, only the rockfish received an impact from the stay-at-home index, and the horse mackerel had an impact from traded volume. The effects were negative on the price in both the positive and negative shocks in the stay-at-home index and volume of trade, respectively.

In the third SOE period, a distinctive influence on the price from the stay-at-home index became apparent among the type of seafood. While positive impacts are seen from the changes in the hours of stay-at-home on horse mackerel and sardine prices, negative but significant impacts are detected on mackerel and Japanese tiger shrimp prices from the shocks in the hours of stay-at-home. This could be due to an increase in the demand for cheap popular fish like horse mackerel and sardine from the household sector in the third SOE while demand for luxury seafood continued to be stagnant as the SOE restricted people from eating at restaurants and demand for luxury seafood from restaurants persisted to be low.

Finally, none of the seafood products were affected by the stay-at-home in the fourth SOE period. This might be implying that the SOE measure no longer had an impact on human mobility as the average hours of stay-at-home became lower in the fourth SOE period.

The distinction in the impact from the SOE measure between the cheap popular and luxury seafood was also apparent in the NARDL short-run coefficient estimations (Table 8). Looking at the stay-at-home indices those were significant at the 5% level, horse mackerel and sardine had a negative impact from the stay-at-home index when the hours of stay-at-home had a decreasing trend in the first SOE period. In contrast, the daily rockfish price had a positive influence from the daily change in the stay-at-home index when the index was declining. Similarly, mackerel price was adversely affected by the negative shocks in the stay-at-home index in the third SOE period while rockfish price was positively affected by the index during the third and fourth SOE periods. These results likely imply that when people began to go out of their homes during the SOE periods, prices of cheap fish received a negative impact from the daily changes in human mobility while luxury fish like rockfish price was impacted positively. This indicates that at least in the short run there was a period where a shift from cheap to luxury seafood occurred as more people began to eat seafood at restaurants as the pandemic calmed down. This contrastive result between the cheap and luxury result is somewhat similar to the findings of Abe et al. [44] suggesting that high-end fish such as red seabream and black throat seaperch are in high demand in luxury restaurants that have been greatly affected by the declaration of a state of emergency, while fish such as Japanese amberjack, a general consumer preference, was seemingly less affected.

**Table 8:** NARDL short-run coefficients and the effects of COVID-19 cases.

	Cheap									Luxury								
	Horse mackerel			Sardine			Mackerel			Shrimp			Rockfish			Sea urchin		
**SOE 1**	**Coef**.		**t-stat**.	**Coef**.		**t-stat**.	**Coef**.		**t-stat**.	**Coef**.		**t-stat**.	**Coef**.		**t-stat**.	**Coef**.		**t-stat**.
ΔVolume+	-0.007	***	-6.610	-0.001		-1.344	-0.005	**	-2.056	0.868		0.668	0.145		0.951	2.138	***	2.703
ΔVolume-	-0.003	**	-2.374	-0.003	***	-3.138	0.005	**	2.544	0.746		0.490	-0.662	**	-2.606	-2.857	***	-3.185
ΔHome+	2.503		0.652	2.546		1.288	0.241		0.188	-156.871	***	-3.226	-77.955	*	-1.775	-66.390		-1.515
ΔHome-	-8.633	***	-2.805	-3.305	**	-2.437	-1.474		-0.847	22.579		0.293	78.818	**	2.299	1.796		0.018
COVID-19	0.478		1.288	0.208		1.306	0.050		0.219	-13.659	**	-2.034	1.279		0.573	4.168		0.467
**SOE 2**	**Coef**.		**t-stat**.	**Coef**.		**t-stat**.	**Coef**.		**t-stat**.	**Coef**.		**t-stat**.	**Coef**.		**t-stat**.	**Coef**.		**t-stat**.
ΔVolume+	-0.005	***	-5.296	-0.006	***	-3.920	-0.001		-0.950	3.615	*	1.991	-0.120		-0.471	0.091		0.178
ΔVolume-	-0.005	***	-5.408	-0.002		-1.119	-0.002	***	-3.175	-1.953		-1.299	0.167		0.664	0.709		1.194
ΔHome+	-6.547		-1.469	0.638		0.144	-2.277		-1.185	-240.785	*	-1.714	0.889		0.012	-109.877		-0.332
ΔHome-	-7.713		-1.406	5.352		1.064	-3.966		-1.480	-15.071		-0.134	-79.747		-1.311	26.116		0.137
COVID-19	0.004		0.142	-0.012		-0.474	0.019		1.239	-1.814	*	-1.908	0.061		0.179	1.301		1.075
**SOE 3**	**Coef**.		**t-stat**.	**Coef**.		**t-stat**.	**Coef**.		**t-stat**.	**Coef**.		**t-stat**.	**Coef**.		**t-stat**.	**Coef**.		**t-stat**.
ΔVolume+	-0.003	***	-3.857	-0.001		-0.863	-0.009	***	-2.993	-1.571		-0.603	0.986	**	2.451	0.461		0.768
ΔVolume-	-0.001		-0.707	-0.003	***	-3.102	-0.009	**	-2.382	2.382		0.956	-0.269		-0.554	-2.254	**	-2.068
ΔHome+	13.459	***	3.558	21.247	***	4.284	0.125		0.023	302.867		1.550	-121.858		-1.413	-101.275		-0.546
ΔHome-	2.837		0.729	6.945		1.273	-10.814	**	-2.262	-157.335		-0.756	215.980	***	2.908	241.229		1.321
COVID-19	0.006		0.161	-0.018		-0.348	-0.006		-0.102	-3.233	**	-2.277	0.300		0.466	-1.038		-0.585
**SOE 4**	**Coef**.		**t-stat**.	**Coef**.		**t-stat**.	**Coef**.		**t-stat**.	**Coef**.		**t-stat**.	**Coef**.		**t-stat**.	**Coef**.		**t-stat**.
ΔVolume+	-0.004	***	-5.334	-0.001		-0.809	-0.001		-0.563	-0.137		-0.093	0.021		0.053	-0.082		-0.109
ΔVolume-	-0.002	**	-2.225	-0.002	**	-2.002	-0.001		-0.445	1.613		1.051	-0.068		-0.183	-1.082		-1.170
ΔHome+	-6.347	*	-1.801	3.043		0.579	-0.323		-0.084	0.273		0.003	-24.806		-0.409	-50.611		-0.346
ΔHome-	0.496		0.141	3.722		0.882	2.718		0.789	-80.655		-0.916	156.919	***	2.706	176.236		1.120
COVID-19	0.009		1.465	-0.007		-0.675	-0.003		-0.371	0.052		0.288	0.137		1.059	0.721	**	2.285

Note:

***, **, and * denote significance at the 1%, 5%, and 10% levels, respectively.

To check the validity of applying the NARDL model, we also conducted the Wald test for short- and long-run asymmetry. The results in Table 9 signify that a prominent long-run asymmetry became evident in the long run during SOE 1 and SOE 3. This is consistent with the long-run estimation where stay at home index mostly became significant during the SOE 1 and SOE 3 periods. Similarly, the short-run asymmetry was also more apparent during the SOE 1 and SOE 3 periods where the stay-at-home index had significant effects in both the cheap and luxury seafood. Hence, these results suggest the importance of applying the NARDL model when the endogenous variable investigated tends to have asymmetric impacts on the dependent variable.

**Table 9:** Wald test for long-run and short-run asymmetry.

Home	SOE 1				SOE 2				SOE 3				SOE 4			
	Long-run		Short-run		Long-run		Short-run		Long-run		Short-run		Long-run		Short-run	
Horse mackerel	4.89	**	2.92	*	0.20		0.20		5.17	**	5.20	***	2.61		2.11	
Sardine	3.24	*	1.72		0.82		2.78	**	4.10	**	6.88	***	0.02		4.00	**
Mackerel	4.88	**	4.88	**	0.60		0.60		4.72	**	8.40	***	1.64		1.64	
Shrimp	4.44	**	2.33		8.00	***	8.00	***	3.48	*	4.00	***	0.72		0.72	
Rockfish	8.82	***	3.52	***	0.72		4.84	***	10.40	***	4.93	***	5.29	**	4.19	***
Sea urchin	0.50		1.04		0.17		2.03	*	1.03		2.90	**	1.08		2.30	*
**Volume**																
Horse mackerel	5.57	**	21.08	***	0.18		0.18		3.54	*	1.87		3.68	*	7.32	***
Sardine	1.84		5.78	***	7.44	***	8.40	***	15.39	***	15.39	***	12.96	***	12.96	***
Mackerel	12.95	***	5.06	***	0.49		2.89	**	0.00		8.57	***	0.01		2.54	*
Shrimp	0.00		4.81	***	8.95	***	5.58	***	5.47	**	6.61	***	1.13		1.13	
Rockfish	8.85	***	5.11	***	8.73	***	8.73	***	7.56	***	5.19	***	0.24		0.24	
Sea urchin	11.61	***	5.61	***	1.83		1.83		5.00	**	2.62	*	0.63		1.72	

***, **, and * denote the significance at the 1%, 5%, and 10% levels, respectively. The numbers in the table represent the F-statistic.

## Conclusions

The COVID-19 pandemic has disrupted human mobility as many countries-imposed restrictions like lockdowns and declared a state of emergency to decrease the number of COVID-19 cases. Such a decrease in human mobility has affected the global agricultural commodity market, including seafood. Japan is one of the world’s most sea fish-consuming countries, and its seafood market was also interrupted due to the SOE restriction at different stages. To understand the effects of the SOE restrictions during the COVID-19 pandemic on the Japanese seafood market, the study investigated how the changes in the hours of stay at home during the SOE periods affected the seafood markets. Since luxury seafood products that are predominantly consumed in restaurants are likely to have more adverse impact compared to cheap popular seafood commonly consumed at homes, the study examined the difference in the effects received on these seafood products from the SOE measure.

Using the NARDL estimation we identified that both the cheap and luxury seafood were adversely affected by the SOE measure during the first SOE period (10 March 2020 to 22 June 2020) in the long run. When hours of stay-at-home were changing, the prices of seafood had a declining trend. However, in the third SOE period (28 March 2021 to 17 July 2021), cheap seafood like horse mackerel and sardine prices had a positive impact from the SOE measure while the luxury seafood price continued to receive a negative impact. The short-run impact also revealed a distinctive influence on prices from the SOE measure between cheap and luxury seafood. When the hours of stay-at-home was decreasing such that when human mobility had an increasing trend during the SOE periods, mackerel (cheap seafood) price was decreasing while rockfish price (luxury seafood) had an increasing impact. This could be indicating that there were times when more people started consuming luxury seafood as they were able to dine at restaurants after the pandemic subsided, shifting their preference from cheap seafood prepared at home to luxury seafood typically enjoyed in restaurants.

In sum, the results of the study indicate that the SOE measure particularly had a negative impact on luxury seafood prices in the long run but there were times in the short run when luxury seafood prices were affected positively by changes in human mobility as the effect of the SOE measure weakens.

The findings of the study imply the importance of supporting the luxury seafood markets depends highly on the restaurant sector, which will likely face adverse impacts when human mobility is restricted. Thus, policymakers need to stabilize the price and provide subsidies for the stakeholders of the luxury seafood market during a pandemic like COVID-19.

The generalization of the findings of this study is subject to certain limitations. The regressed variables used in this study (based on uniform data availability during the four SOE periods) may not be sufficient to capture the entire picture.

For instance, due to daily data, it was not possible to collect other trustful data like people’s behaviors in consuming seafood, the ease of making online seafood purchases, and the convenience of home delivery, though these variables might influence the fluctuation of the market prices for seafood where we assume that all such information is included in the price. However, the current study can contribute to understanding the impacts of COVID-19 on cheap and luxury seafood prices at different SOE periods. Although the outcomes of our NARDL model are only rough indicators, the study results can contribute to the debate on the short-run and long-run impacts, and hence, there is scope for policy planners to adapt the model and change the variables according to necessity.

## Supporting information

S1 Fig CUSUM and CUSUM square tests. (DOCX)

## References

[1] The Organisation for Economic Co-operation and Development (OECD). 2020. *Fisheries*, *aquaculture and Covid-19*: *Issues and policy responses*, *OECD Policy Responses to Coronavirus*. http://www.oecd.org/coronavirus/policy-responses/fisheries-aquaculture-and-covid-19-issues-and-policy-responses-a2aa15de/. Accessed on March 11, 2022.

[2] Food and Agricultural Organization of the United Nations (FAO) 2020. *How is Covid-19 outbreak impacting the fisheries and aquaculture food systems and what can FAO do*. FAO Fisheries and Aquaculture Department. http://www.fao.org/3/cb1436en/cb1436en.pdf. Accessed on March 11, 2022.

[3] Food and Agriculture Organization (FAO). 2020. *The state of world fisheries and aquaculture—Sustainability in Action*, Rome. 206 pp. http://www.fao.org/documents/card/en/c/ca9229en/. Accessed on March 11, 2022.

[4] Makino M. 2011. Fisheries Management in Japan: its institutional Features and Case Studies. Fish & Fisheries series book 34. Springer, pp 1–200.

[5] United States Department of Agriculture (USDA). 2020. *Japan*: *COVID-19 Impacts on Food Distribution in Japan*. April 09, 2020. https://www.fas.usda.gov/data/japan-covid-19-impacts-food-distribution-japan. Accessed on March 11, 2022.

[6] Japan International Research Center for Agricultural Sciences (JIRCAS). 2020. *45*. *The Impact of COVID-19 on Fisheries and Aquaculture*. https://www.jircas.go.jp/en/program/program_d/blog/20200623. Accessed on March 10, 2022.

[7] SeaFoodSource. 2020. *Toyosu sales figures reveal crippling effect of COVID-19 on Japan’s seafood market*. https://www.seafoodsource.com/news/supply-trade/toyosu-sales-figures-reveal-crippling-effect-of-covid-19-on-japan-s-seafood-market. Accessed on March 10, 2022.

[8] Ahmadiara E. 2020. Possibility of Faecal-Oral transmission of novel coronavirus (SARS-CoV-2) via consumption of contaminated foods of animal origin: a hypothesis. *Journal of Food Quality & Hazards Control*, 7(1): 2–3. http://jfqhc.ssu.ac.ir/article-1-662-en.html.

[9] Bassett HR, Lau J, Giordano C, Suri SK, Advani S, Sharan S. 2021. Preliminary lessons from COVID-19 disruptions of small-scale fishery supply chains. *World Development*, 143, 105473. doi: 10.1016/j.worlddev.2021.105473 36567900 PMC9758721

[10] White ER, Froehlich HE, Gephart JA, Cottrell RS, Branch TA, Agrawal Bejarano R., et al. 2021. Early effects of COVID-19 on US fisheries and seafood consumption. *Fish Fisheries*, 22(1): 232–239. doi: 10.1111/faf.12525 33362433 PMC7753393

[11] Watanabe T, Yabu T. 2021. Japan’s voluntary lockdown. *PLoS ONE*, 16:e0252468. doi: 10.1371/journal.pone.0252468 34111163 PMC8191912

[12] Ritchie H, Mathieu E, Rodés-Guirao L, Appel C, Giattino C, Ortiz-Ospina E. et al. 2020. Coronavirus Pandemic (COVID-19). Our World Data. https://ourworldindata.org/covid-google-mobility-trends. Accessed on June 22, 2023.

[13] Nishijima C, Miyagawa N, Tsuboyama-Kasaoka N, Chiba T, Miyachi M. 2021. Association between lifestyle changes and at-home hours during and after the state of emergency due to the COVID-19 pandemic in Japan. *Nutrients*, 13(8):2698. doi: 10.3390/nu13082698 34444858 PMC8398728

[14] Kitamura Y, Karkour S, Ichisugi Y, Itsubo N. 2020. Evaluation of the economic, environmental, and social impacts of the COVID-19 pandemic on the Japanese tourism industry. *Sustainability*, 12(24):10302. doi: 10.3390/su122410302

[15] Dietrich S, Giuffrida V, Martorano B, Schmerzeck G. 2022. COVID-19 policy responses, mobility, and food prices. *American Journal of Agricultural Economics*, 104(2): 569–588. doi: 10.1111/ajae.12278

[16] Hillen J. 2021. Online food prices during the COVID-19 pandemic. *Agribusiness*, 37: 91–107. doi: 10.1002/agr.21673 33362338 PMC7753706

[17] Ruan J, Cai Q, Jin S. 2021. Impact of COVID-19 and Nationwide Lockdowns on Vegetable Prices: Evidence from Wholesale Markets in China. *American Journal of Agricultural Economics*, 103(5): 1574–1594. doi: 10.1111/ajae.12211 33821009 PMC8014438

[18] Cranfield JAL. 2020. Framing consumer food demand responses in a viral pandemic. *Canadian Journal of Agricultural Economics/Revue canadienne d’agroeconomie*, 68(2): 151–156. doi: 10.1111/cjag.12246

[19] Shin Y, Yu B, Greenwood-Nimmo M. 2014. Modelling Asymmetric Cointegration and Dynamic Multipliers in a Nonlinear ARDL Framework. In: Sickles R., Horrace W. (eds) Festschrift in Honor of Peter Schmidt. Springer, New York, NY.

[20] Pesaran MH, Shin Y, and Smith RJ. 2001. Bounds testing approaches to the analysis of level relationships. *Journal of Applied Econometrics*, 16(3): 289–326. doi: 10.1002/jae.616

[21] Pesaran MH, Shin Y. 1998. An autoregressive distributed lag modelling approach to cointegration analysis. In: Strom S (ed) Econometrics and economic theory: the Ragnar Frisch centennial symposium. Cambridge University Press, Cambridge.

[22] Aruga K. 2022. Effects of the COVID-19 Pandemic on the Tokyo Wholesale Tuna Market. *Journal of International Food & Agribusiness Marketing*, doi: 10.1080/08974438.2022.2152518

[23] Jannat A, Aruga K, Furuya J, Iiyama M. 2022. Investigating the Impact of International Markets on Imported and Exported Non-Cereal Crops in Bangladesh. *Agriculture*, 12(6), 833. doi: 10.3390/agriculture12060833

[24] Van Treeck T. 2008. Asymmetric income and wealth effects in a non-linear error correction model of US consumer spending. Working paper no. 6/2008, Macroeconomic Policy Institute in the Hans-Böckler Foundation, Düsseldorf.

[25] Delatte AL, Lopez-Villavicencio A. 2012. Asymmetric exchange rate pass-through: Evidence from major countries. *Journal of Macroeconomics*, 34(3): 833–844. doi: 10.1016/j.jmacro.2012.03.003

[26] Aruga K. 2021. Changes in human mobility under the covid-19 pandemic and the Tokyo fuel market. *Journal of Risk and Financial Management*, 14, 163. doi: 10.3390/jrfm14040163

[27] Aruga K, Islam MM, Jannat A. 2021. Does staying at home during the covid-19 pandemic help reduce CO2 emissions? *Sustainability*, 13(15), 8534. doi: 10.3390/su13158534

[28] Reardon T, Bellemare MM, Zilberman D. 2020. How COVID-19 may disrupt food supply chains in developing countries. IFPRI Book chapters: 78–80. Accessed on March 7, 2022.

[29] Akter S. 2020. The impact of COVID-19 related stay-at-home restrictions on food prices in Europe: findings from a preliminary analysis. *Food Security*, 12(4): 719–725. doi: 10.1007/s12571-020-01082-3 32837638 PMC7340765

[30] Rathod NB, Elabed N, Özogul F, Regenstein JM, Galanakis CM, Aljaloud SO, et al. 2022. The impact of COVID-19 pandemic on seafood safety and human health. *Frontiers in Microbiology*, 13: 875164. doi: 10.3389/fmicb.2022.875164 35814679 PMC9257084

[31] Ahmed N, Khan D, Oláh J, Popp J. 2023. A comparative study of the policy response to COVID-19 in the ASEAN region: A dynamic simulated ARDL approach. *PLoS ONE*, 18(1): e0276973. doi: 10.1371/journal.pone.0276973 36701393 PMC9879420

[32] Sugimoto A, Roman R, Hori J, Tamura N, Watari S, Makino M. 2022. “How has the ’customary nature’ of Japanese fisheries reacted to Covid-19? An interdisciplinary study examining the impacts of the pandemic in 2020.” *Marine Policy*, 138. doi: 10.1016/j.marpol.2022.105005 35185265 PMC8847101

[33] Errhalt G. 2020. COVID-19 harms Japanese fisheries despite active fleet. Global Fishing Watch, September 13. https://globalfishingwatch.org/news-views/covid-19-japanese-fisheries/. Accessed on March 29, 2023.

[34] World Population Review. 2022. Tokyo Population 2022. https://worldpopulationreview.com/world-cities/tokyo-population. Accessed on March 11, 2022.

[35] Giovanni O, Silvana T. 2007. "The Timing of Monetary Policy Shocks," *American Economic Review*, *American Economic Association*, 97(3): 636–663. doi: 10.1257/aer.97.3.636

[36] Google LLC. (2022). *Google COVID-19 community mobility reports*. https://www.google.com/covid19/mobility/. Accessed on February 6, 2022.

[37] Zivot E, Andrews DWK. 1992. Further evidence on the great crash, the oil-price shock and the unit-root hypothesis. *Journal of Business Economic Statistics*, 10(3): 251–270. 10.1080/07350015.1992.10509904.

[38] Demirhan H. 2020. dLagM: An R package for distributed lag models and ARDL bounds testing. *PLoS ONE*, 15(2): e0228812. doi: 10.1371/journal.pone.0228812 32084162 PMC7034805

[39] Breusch TS. 1978. “Testing for Autocorrelation in Dynamic Linear Models.” *Australian Economic Papers*, 17: 334–355. doi: 10.1111/j.1467-8454.1978.tb00635.x

[40] Godfrey GL. 1978. Testing against general autoregressive and moving average error models when the regressors include lagged dependent variables. *Econometrica*, 46(6): 1293–1301. doi: 10.2307/1913829

[41] Breusch TS, Pagan AR. 1979. A simple test for heteroscedasticity and random coefficient variation. *Econometrica*, 47 (5): 1287–94.

[42] Kawamoto D. 2020. COVID-19 Disruption Puts Toyosu’s Top-Shelf Fish in Easy Reach. https://www.nippon.com/en/japan-topics/g00855/?fbclid=IwAR3_5PjsyV0Jtt1nDvqHgtM9Qs_GBTyWiqw3OOgH2Bl4LFBM-TEk3KKPY3M. Accessed on March 28, 2023.

[43] Kobayashi M. 2022. The COVID-19 impacts and challenges to achieving sustainability in Japan’s fisheries and aquaculture. *Marine Policy*, 143. doi: 10.1016/j.marpol.2022.105161 35945917 PMC9352237

[44] Abe K, Ishimura G, Baba S. et al. 2022. Evaluating the impact of COVID-19 on ex-vessel prices using time-series analysis. *Fisheries Science*, 88: 191–202. doi: 10.1007/s12562-021-01574-x 35095191 PMC8784210

[45] Tokyo Metropolitan Government. 2022. *Updates on COVID-19 in Tokyo*. https://stopcovid19.metro.tokyo.lg.jp/en. Accessed on March 12, 2022.

[46] Metropolitan Central Wholesale Market (MCWM). 2022. *Metropolitan central wholesale market daily report*. https://www.shijou-nippo.metro.tokyo.lg.jp/index.html. Accessed on February 9, 2022.

