# Supplementary material for: The impacts of COVID-19 on seafood prices in Japan: A comparison between cheap and luxury products
Source: PLoS One. 2023 Oct 5;18(10):e0291395. doi: 10.1371/journal.pone.0291395 (PMC10553297; doi:10.1371/journal.pone.0291395)
Supplement: S1 Fig — (DOCX) [file pone.0291395.s001.docx]

1. SOE 1

1. SOE 2

1. SOE 3

1. SOE 4

S1 Fig. (a) CUSUM and CUSUM square tests for horse mackerel

1. SOE 1

1. SOE 2

1. SOE 3

1. SOE 4

S1 Fig. (b) CUSUM and CUSUM square tests for sardine

1. SOE 1

1. SOE 2

1. SOE 3

1. SOE 4

S1 Fig. (c) CUSUM and CUSUM square tests for mackerel

1. SOE 1

1. SOE 2

1. SOE 3

1. SOE 4

S1 Fig. (d) CUSUM and CUSUM square tests for Japanese tiger shrimp

1. SOE 1

1. SOE 2

1. SOE 3

1. SOE 4

S1 Fig. (e) CUSUM and CUSUM square tests for channel rockfish

1. SOE 1

1. SOE 2

1. SOE 3

1. SOE 4

S1 Fig. (f) CUSUM and CUSUM square tests for sea urchin
